# Association of Weight Changes by Three Days after Birth and Mortality and/or Severe Neurological Injury in Preterm Infants < 29 Weeks Gestational Age: A Multicenter Cohort Study

**DOI:** 10.3390/children9020276

**Published:** 2022-02-17

**Authors:** Carlos Zozaya, Khalid Aziz, Nalini Singhal, Xiang Y. Ye, Christine Drolet, Julie Emberley, Kyong-Soon Lee, Vibhuti S. Shah, on behalf of the Canadian Neonatal Network (CNN) Investigators

**Affiliations:** 1 Department of Paediatrics, Mount Sinai Hospital, Toronto, ON M5G 1X5, Canada; carlos.zozaya@madrid.salud.org; 2 Department of Pediatrics, University of Alberta, Edmonton, AB T6G 1C9, Canada; khalid.aziz@ualberta.ca; 3 Department of Pediatrics, University of Calgary, Calgary, AB T2N 1N4, Canada; nalini.singhal@albertahealthservices.ca; 4 Maternal-Infant Care (MiCare) Research Centre, Mount Sinai Hospital, Toronto, ON M5G 1X6, Canada; philip.ye@sinaihealth.ca; 5 Centre Mère-Enfant Soleil, Centre Hospitalier Universitaire de Québec, Université Laval, Quebec City, QC G1V 4G2, Canada; christine.drolet.med@ssss.gouv.qc.ca; 6 Department of Pediatrics, Janeway Children’s Health and Rehabilitation Center, Memorial University of Newfoundland, St. John’s, NL A1B 3V6, Canada; julie.emberley@med.mun.ca; 7 Division of Neonatology, The Hospital for Sick Children, Toronto, ON M5G 1X8, Canada; kyong-soon.lee@sickkids.ca

**Keywords:** preterm-infant, weight, mortality, neurological injury

## Abstract

Objective: This study aimed to determine the range of weight loss, at 3 days postnatal age, associated with the lowest risk of mortality/short-term morbidity in preterm infants <29 weeks gestational age (GA). Study design: This multicenter retrospective cohort study employed data from the Canadian Neonatal Network database. The primary outcome was a composite of mortality and/or severe neurological injury. Multivariable quadratic and linear regression models which adjusted for potential confounders were built. Results: A total of 9275 preterm infants (median GA 26, IQR 25, 28 weeks) were included. The optimal weight change range at day three, after adjustment for potential confounders for the primary outcomes, was −15 to −8.9%. Conclusions: There is a ‘U’-shaped relationship between weight change from birth to day three and mortality and/or severe neurological injury. Interventional studies, which target weight loss within the range found in this study and evaluate the impact on neonatal outcomes, are needed to corroborate our findings.

## 1. Introduction

A reduction in total body water content, due to the contraction of the extracellular compartment, is part of the transition from intrauterine to extrauterine life and is reflected by initial weight loss [1,2,3]. Studies have suggested that the degree of weight loss soon after birth is important and is associated with both mortality and morbidity rates. Excessive weight loss has been associated with intraventricular hemorrhage (IVH) [4], whereas failure to lose weight has been associated with the combined outcome of death or bronchopulmonary dysplasia (BPD) and patent ductus arteriosus (PDA) [5,6]. One postulated mechanism for the increased incidences of both BPD and PDA involves increased fluid retention in the pulmonary interstitial tissue, resulting in reduced lung compliance and a higher need for respiratory support such as longer duration of mechanical ventilation and oxygen therapy, resulting in potential lung injury and occurrence of BPD [5]. The prescription of total fluid and sodium intake could modulate this adaptive process [7]. Excess water intake may result in an iatrogenic fluid overload, especially during the first 48 h when there is obligate oliguria [8]. On the other hand, preterm infants are at a high risk of elevated transdermal water losses due to the immaturity of the skin barrier, which may lead to hypernatremic dehydration [1].

As a consequence of these concerns, individualized fluid management is common practice in neonatal intensive care units (NICUs), usually based on daily weight changes, fluid intake and output, and trends in serum sodium levels [1]. However, the optimal weight loss (or gain) during the initial postnatal adaption period is unknown [9]. We therefore lack evidence-based targets to guide our fluid and electrolyte management. Evaluation of the association between early weight changes and later clinical outcomes would be the first step towards determining optimal weight change ranges/targets, and may be used to design future interventional studies. We hypothesized that there would be a ‘U’-shaped relationship between early weight changes and neonatal outcomes, based on the previous literature findings which showed harmful effects of both excessive weight loss and no weight loss/weight gain [4,5,6]. The aim of this study was to determine the range of weight change by day three of postnatal age that is associated with the lowest risk of mortality and/or severe neurological injury (SNI). In addition, the secondary outcomes of BPD and PDA were evaluated.

## 2. Methods

### 2.1. Study Design and Population

This was a retrospective cohort study of preterm infants born between 22^0^ and 28^6^ weeks of gestational age (GA), admitted to 31 NICUs in the Canadian Neonatal Network (CNN) [10], from 1 January 2010 to 31 December 2017. Neonates who were moribund on admission or died before three days of age, those with major congenital malformation or chromosomal abnormalities, and those with missing data on weight at birth or at three days of age were excluded. The study was approved by the Mount Sinai Hospital Research Ethics Board (20-0196-C) and permission was obtained from the Executive Committee of the CNN prior to initiation of the study.

### 2.2. Source of Data

At all CNN-affiliated sites, demographic and outcome data are collected from the patients’ charts by trained research assistants using a computerized data entry program, according to the standardized outcome definitions [11]. The database is reported to have high reproducibility and internal consistency, and represents the majority of infants admitted to tertiary NICUs in Canada [12].

### 2.3. Exposures and Outcomes Variables

The exposure of interest in this study was the weight change from birth to day three of postnatal age, expressed as a percentage of birth weight (delta_W3). In the CNN database, the day of birth is considered day 1 [11]. Mortality was defined as death before discharge from the NICU. Severe neurological injury was defined as the presence of either IVH with ventricular enlargement, or periventricular changes consistent with hemorrhage or infarction (with or without IVH). This descriptive definition is consistent with the historical nomenclature of grade III or IV IVH, or “severe IVH” [13]. Bronchopulmonary dysplasia is defined as the need for oxygen or respiratory support at 36 weeks postmenstrual age at discharge, or at the time of transfer to a level II NICU [14]. Patent ductus arteriosus was defined by diagnosis via echocardiogram, and/or clinical signs and symptoms [11].

The primary outcome was a composite of mortality and/or SNI. The secondary outcomes were the individual components of the composite outcome, BPD, and PDA.

### 2.4. Other Data Collection

Maternal and infant baseline characteristics were collected. Gestational age was estimated using a hierarchy of in-vitro fertilization date, last menstrual date, early antenatal ultrasound dating, obstetric estimate, and neonatal estimate in that sequence. ‘Small for gestational age’ (SGA) was defined as a birth weight <10th percentile for GA and sex, as per the Kramer growth charts specific for the Canadian population [15]. Antenatal steroid therapy was categorized as any (complete or partial dosing), or none. Other variables collected included maternal diabetes and hypertension, mode of delivery, multiple or singleton birth, chest compressions at birth, Apgar score at 5 min, the score for neonatal acute physiology II (SNAP-II) [16], and inborn or outborn status.

### 2.5. Statistical Analysis

The study population was summarized using descriptive statistical methods. We classified the weight change, delta_W3, into nine groups from <−20% to ≥15% in 5% increments. To examine the association between the infants’ characteristics and weight change (%), infant characteristics were compared among the nine weight change (%) groups, using the chi-square test for categorical variables, and the ANOVA (F test) or Kruskal-Wallis test as appropriate for continuous variables.

To examine whether a ‘U’-shaped relationship existed between the rate of outcomes and the weight change from birth to day three (delta_W3), non-linear regression analyses using the quadratic model a(delta_W3)^2^ + b(delta_W3) + c were conducted to fit the outcome rates. If the coefficient of the quadratic term ‘a’ was significantly greater than 0, it implied a significant ‘U’-shaped relationship between the rate of outcomes and the weight change. Multivariable logistic regression quadratic models were also conducted to further determine the ‘U’-shaped relationship between the binary outcomes and weight change, adjusting for potential confounders identified in the univariate analysis or based on clinical relevance. The potential confounders were GA, SGA, sex, SNAP-II score, outborn status, Cesarean section, antenatal steroid use, and chest compressions at birth. The generalized estimating equation (GEE) approach was used for the regressions to account for the clustering of infants within NICUs. If a ‘U’-shaped relationship was found, the minimum point (C) (95% confidence interval [CI]) of the curve was estimated. Then, the corresponding optimal region (C − d1, C + d2) was determined, starting from the minimum point (C) by searching the first d1 (>0) and d2 (>0) such that the risk of an adverse outcome was significantly higher in the regions <C − d1 and ≥C + d2 compared to the region (C − d1, C + d2). If no significant “U”-shaped relationship between an outcome and weight change by day three was found after adjustment, we then examined if there was a linear relationship instead.

Data management and all statistical analyses were performed using the Statistical Analysis Systems (SAS) 9.4 (SAS Institute, Cary, NC, USA) and R 4.0.0 (R Foundation for Statistical Computing, Vienna, Austria). A two-sided *p*-value of <0.05 was considered statistically significant.

## 3. Results

**Figure 1:**
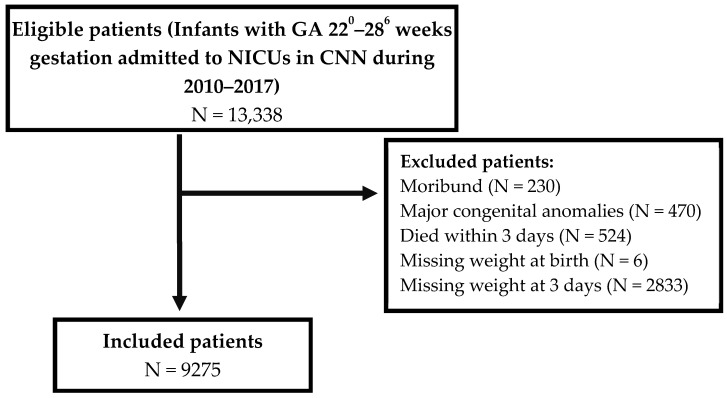
Flow chart of study population.

**Figure 2:**
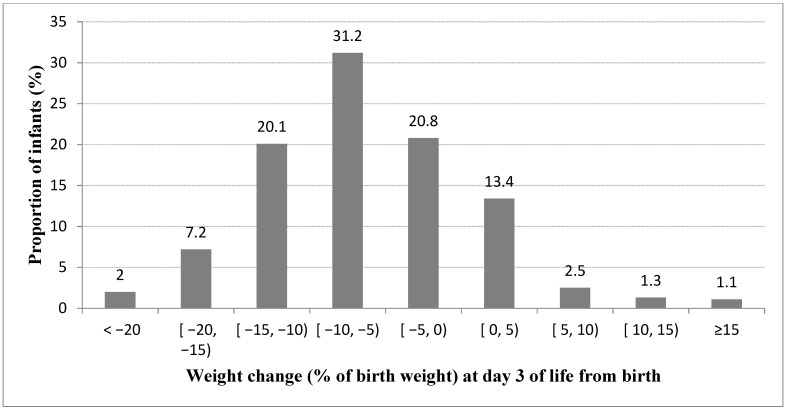
Distribution of weight change (expressed as % of birth weight) by day three of age.

Among 13,338 eligible infants admitted at <29 weeks GA, 9275 (69.5%) fulfilled the inclusion criteria (Figure 1). The distribution of percentage weight change from birth to day three for the included infants is presented in Figure 2. Most patients (81.7%) lost weight within the first three days after birth. The median weight change by day three was −6.8% (IQR −11 to −2.2). Demographic and clinical characteristics of the included patients are summarized in Table 1. There were significant differences regarding perinatal characteristics depending on the weight change by day three. GA was lower and outborn status was more frequent at both ends of the spectrum of weight change than in the middle range. Cesarean section, SGA, need for chest compressions at birth, and a SNAP-II score >20 were more common in infants who gained weight from birth to day three. Any antenatal steroid exposure was less common, and weight at birth was lower in infants who gained weight within the first three days. Except for a statistically significant difference in outborn status, among infants with missing weight change data when compared to those without missing weight change data at day three of age, no differences were noted in other maternal and neonatal characteristics (Table 2).

**Figure 3:**
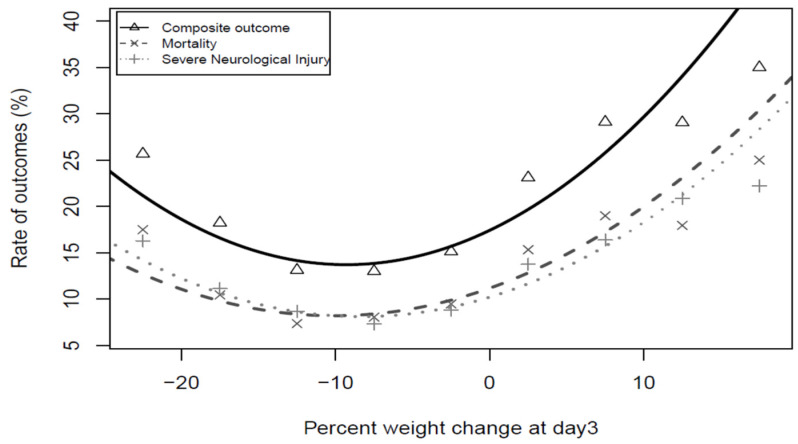
Mortality and/or SNI, mortality and SNI unadjusted frequencies plotted in the fitted quadratic curve.

The univariate analysis showed that the primary outcome and all secondary outcomes (Table 3) were significantly different depending on the direction and degree of weight change from birth to day three of age. A ‘U’-shaped relationship was demonstrated for the primary composite outcome of mortality and/or SNI, after adjustment for GA, SGA, sex, SNAP-II score > 20, outborn status, Cesarean section, any antenatal steroid use, and chest compressions at birth (Figure 3). Then, the minimum point of each curve was calculated (Table 4). Finally, the corresponding optimal ranges associated with the lowest risk of the outcomes were determined. The optimal range identified for the composite outcome and mortality was [−15 to −9%), while for SNI, the optimal range was [−13 to −9%) (Table 5 and Table 6).

For BPD and PDA, the coefficient of the quadratic term ‘a’ was not significant, implying no significant ‘U’-shaped association after adjustment. Further examinations using linear regression models adjusting for potential confounders were conducted. No significant linear relationship between either of these two outcomes and the weight change by day three was observed either.

## 4. Discussion

### 4.1. Summary of the Main Findings

In this large national cohort of preterm infants born at <29 weeks GA, a ‘U’-shaped relationship was observed for weight change between birth and day three of age and the composite outcome of mortality and/or SNI, as well as for each of these two outcomes individually.

### 4.2. Comparison with Previous Literature

Previous retrospective cohort studies have reported data on the relationship between weight changes and mortality, IVH, BPD, and PDA in preterm infants [5,17,18,19,20,21,22,23]. In keeping with our results, Aksoy et al. [22], in a single center cohort study including 126 infants with birth weight <1000 g, reported that the overall mortality, and mortality in the first 7 days, were associated with both no weight loss and excessive weight loss by day three of age. These adverse outcomes were higher in patients with weight loss <3% or >12% (first and fourth quartiles, respectively) by day three compared to those who fell between [22]. Wadhawan et al. [23] conducted the largest study to date on the relationship between weight loss and neonatal outcomes in preterm infants < 1000 g and 24 to 29 weeks GA (N = 9461), using data from the NICHD Neonatal Research Network database [24]. Infants were divided into two groups depending on weight change over the first ten days after birth: no weight loss/weight gain versus any weight loss. Infants who did not lose weight at any point in the first ten days of life had significantly higher mortality, which is in agreement with our findings. Regarding SNI, the literature has shown discordant results as SNI has been associated with both no postnatal weight loss/weight gain, as well as with excessive weight loss [21,22]. Verma et al. [21] reported a protective borderline association between a higher weight loss by day five and a lower risk of any grade of IVH, while Aksoy et al. [22] reported an association between a higher weight loss by day three and an increased incidence of any grade of IVH. These contradictory findings may be supportive of the ‘U’-shaped relationship noted in our study, which utilized a larger sample size. In other studies by Lim et al. [19] and Lee et al. [20], no significant association between weight change on day three and IVH was noted, however, the limitations of these studies include small sample sizes resulting in a lack of statistical power.

A failure to lose weight during the postnatal transition has been associated with an increased risk of BPD and PDA [5,21,23]. Wadhawan et al. [23] reported that infants who lost weight at any time between birth and ten days of age had a lower risk of death or BPD compared to those who did not. Similar findings have been reported by Oh et al. [5]. In a secondary analysis of infants in a randomized clinical trial, there was a higher odds ratio of death/BPD, with a lower maximum weight loss within the first ten days after birth [5,25]. However, in our study, no association was found between weight loss and BPD. This difference could be due to the evaluation of weight changes only during the first three days in our study compared to other studies that have evaluated weight change over the first ten days. Our findings are consistent with studies by Verma et al. [21] and Aksoy et al. [22], who also found no association with BPD when weight change was evaluated up to day three.

In summary, the optimal weight change that is associated with a reduction in mortality and short-term neonatal outcomes remains elusive.

### 4.3. Biological Plausibility

Postnatal adaptation after birth includes a contraction of the extracellular fluid volume at the expense of the interstitial compartment, with a shift of water from the extracellular to the intracellular compartment and an overall negative water and sodium balance through insensible water losses and diuresis [1,2]. Three phases have been described during postnatal adaptation [26]. An initial oliguric phase within the first 48 h is followed by postnatal diuresis/natriuresis until 5–7 days of age. Of particular note, urine output does not increase or decrease in response to a higher or lower fluid intake during the first two phases [1,27]. Preterm infants have the ability to dilute urine [28], however, immediately after birth, the low glomerular filtration rate limits their ability to excrete free water if excess fluid is provided during the pre-diuretic phase [27,28,29,30,31]. Thus, preterm infants are at risk of an iatrogenic fluid overload, especially before diuresis peaks. A Cochrane systematic review including five randomized controlled trials conducted between 1980 and 2000 demonstrated that fluid restriction was associated with a decreased risk of PDA and necrotizing enterocolitis, without increasing the risk of dehydration [7]. Interestingly, no effect was seen on either mortality or SNI. However, some of these studies were conducted in the pre-surfactant era, and the definitions of fluid restriction and the duration of the intervention in terms of numbers of days after birth varied widely among studies, making comparisons with our results difficult.

Maximum weight loss occurs during the second phase to approximately 11–13% of birth weight around 5–6 days of age in preterm infants <29 weeks [3,17]. During the first phase, the period most likely represented in our study, weight loss occurs mainly through the insensible route. Transcutaneous losses make up most of the insensible water losses and are greater the lower the GA at birth, but also the lower the postnatal age [32,33]. These losses peak at around 48 h and then decrease even in the most preterm infants, as the stratum corneum of the epidermidis matures [34]. It is during this time that the risk of hypernatremic dehydration is higher [1,35]. Hypernatremia (serum sodium level >150 mmol/L) and sodium fluctuations >13 mEq/L during the first three days have been associated with an increased risk of IVH and parenchymal injury in this population [4,19].

### 4.4. Strength and Limitations

The strengths of our study are a large sample size and the high quality of data utilized for analysis [12]. The statistical approach used in this study allowed us to explore and confirm the existence of a ‘U’-shaped relationship between weight loss by day three and relevant neonatal outcomes, which had not been explored in previous studies. In this study, only weight change by day three was evaluated. Unfortunately, we lacked data regarding weight beyond three days of age to allow comparison with other studies that examined weight changes up to ten days of age. On the other hand, as explained above, we believe there are physiological reasons to consider the first three days (roughly coincident with the prediuretic phase and the time when the skin is more immature) as the highest risk period for both dehydration and fluid overload. Additionally, the exclusion of 21.2% of eligible infants in this analysis due to the unavailability of data on weight either at birth or to a larger extent, day three of life, is another limitation, although no major differences in baseline characteristics were found. Data regarding total fluid intake and types of fluid given (i.e., normal saline boluses in the sicker infants), and plasma sodium concentration were also lacking. These data would have been useful to further explore potential mechanisms to explain our findings (i.e., severe hypernatremia leading to SNI, or extensive fluid resuscitation driving higher mortality and neurological injury in infants who did not lose weight).

### 4.5. Implications for Clinical Practice and Research

The ‘U’-shaped relationship described in this study between weight change and outcomes is hypothesis-generating, whereas causality needs to be proven. Clinical trials exploring fluid management strategies or targets could be undertaken to evaluate whether the important outcomes of mortality or SNI are impacted, using weight loss as an intermediate variable since fluid intake and output will influence weight gain or loss. In addition, further prospective observational studies could (1) examine the risk factors associated with weight changes out of the optimum range identified in this study; (2) explore the range of weight changes associated with a lower mortality and morbidity at the end of the diuretic phase (day 5–7); (3) explore the optimal time for recovery to birth weight during the post-diuretic phase.

## 5. Conclusions

We observed a ‘U’-shaped relationship between weight loss from birth to day three and mortality and/or SNI in preterm infants born at <29 weeks GA. Interventional studies targeting weight loss within the optimal range found in our study, with evaluation of the impact neonatal outcomes, are needed to corroborate these findings.

## Figures and Tables

**Table 1:** Comparison of maternal and neonatal characteristics among the groups of weight change (% of birth weight) between birth and day three.

Variables *	<−20	[−20 to −15)	[−15 to −10)	[−10 to −5)	[−5 to 0)	[0 to 5)	[5 to 10)	[10 to 5)	≥15	*p*-Value **
Infants (N) **	183	669	1902	2891	1930	1246	237	117	100	
ANS (any)	91.7 (165/180)	89.4 (589/659)	90.7 (1702/1877)	90.5 (2580/2852)	89.1 (1690/1897)	86.1 (1050/1220)	82.5 (193/234)	79.5 (89/112)	88 (88/100)	<0.001 ‡
Sex (male)	54.6 (100/183)	49.5 (331/669)	51.1 (971/1901)	55.8 (1611/2889)	54.2 (1043/1926)	55 (683/1243)	53.2 (125/235)	53.9 (63/117)	52 (52/100)	0.04
Outborn	17.5 (32/183)	15.1 (101/669)	15 (285/1902)	13.2 (382/2891)	14.3 (275/1930)	15.7 (196/1246)	21.1 (50/237)	19.7 (23/117)	19 (19/100)	0.01
CS	56.8 (104/183)	49.9 (333/667)	53.2 (1008/1896)	56.6 (1635/2889)	61.4 (1184/1927)	60.4 (752/1246)	74.3 (176/237)	71.8 (84/117)	67 (67/100)	<0.001 ‡
GA (weeks)	26 (24, 27)	26 (25, 27)	27 (25, 28)	27 (25, 28)	27 (25, 28)	26 (25, 27)	26 (25, 27)	26 (25, 27)	26 (25, 27)	<0.001 ‡
BW (g)	960 (760, 1156)	940 (760, 1130)	976 (795, 1140)	940 (771, 1130)	890 (740, 1060)	820 (670, 1000)	740 (593, 930)	730 (600, 940)	725 (584.5, 850)	<0.001 ‡
SGA	3.3 (6/183)	3.7 (25/669)	3.1 (59/1901)	6.1 (176/2890)	9.6 (184/1927)	14.6 (182/1245)	27.5 (65/236)	30.8 (36/117)	37 (37/100)	<0.001 ‡
SNAP-II score > 20	32.8 (60/183)	27.1 (181/668)	23 (436/1899)	23.7 (685/2890)	27.5 (530/1926)	35 (436/1246)	49.6 (117/236)	53 (62/117)	47 (47/100)	<0.001 ‡
CC	4.9 (9/183)	7.8 (52/669)	5.2 (99/1902)	5 (144/2890)	5.7 (110/1929)	6.7 (83/1246)	13.9 (33/237)	10.3 (12/117)	15 (15/100)	<0.001 ‡

* Data are presented as median (IQR) or % (n/N) depending on the variable. ** The reported *p*-Values are based on comparisons among weight change groups using the Chi-square test for categorical variables and the Wilcoxon rank sum test for continuous variables. ‡ Trend tests were conducted using the Cochran-Armitage trend test or quantile regression (the symbol ‡ indicates a significant increasing/decreasing trend: *p* < 0.05). A significant *p*-Value derived from the Chi-square test implied that there may be a significant association between the studied characteristic and weight change, while the trend test showed whether there was a significant trend or not. ANS = antenatal steroids; BW = birth weight; CC = chest compressions; CS = Cesarean section; GA = gestational age; IQR = interquartile range; N = number of eligible infants; n = number of infants with outcome data; SD = standard deviation; SGA = small for gestational age; SNAP-II = score for neonatal acute physiology II. ** The denominator may vary for different characteristics due to missing data.

**Table 2:** Maternal and neonatal characteristics between those with and without missing weight change (% of birth weight) data at day three of age.

Variables *	Weight at Day3 Available	Weight at Day3 Missing	*p*-Value
Infants (N) **	9275	2833	
Antenatal corticosteroids (any vs. none), % (n/N)	89.2 (8146/9131)	89.4 (2426/2714)	0.8
Gestational age (weeks), median (IQR)	26 (25, 28)	26 (25, 28)	0.6
Weight at birth (g), median (IQR)	910 (740, 1100)	909 (738, 1100)	0.6
Sex (male), % (n/N)	53.8 (4979/9263)	55.2 (1562/2829)	0.2
Small for gestational age, % (n/N)	8.3 (770/9268)	8.7 (245/2829)	0.6
SNAP-II score > 20, % (n/N)	27.6 (2554/9265)	29.5 (814/2763)	0.05
Outborn status, % (n/N)	14.7 (1363/9275)	21.4 (604/2826)	<0.001
Chest compression, % (n/N)	6 (557/9273)	6.6 (185/2209)	0.3

* The *p*-Values are based on comparisons between the two groups using the Chi-square test for categorical variables and a Student’s *t* test or Wilcoxon rank sum test as appropriate for continuous variables. IQR = interquartile range; N = number of eligible infants; n = number of infants with outcome data. ** The denominator may vary for different characteristics due to missing data.

**Table 3:** Association of weight change at day three of age (% of birth weight) and short-term neonatal outcomes.

Weight Change at Day 3 (% of Weight at Birth)										*p*-Value **
Outcomes *	<−20	[−20 to −15)	[−15 to −10)	[−10 to −5)	[−5 to 0)	[0 to 5)	[5 to 10)	[10 to 15)	≥15	
Infants (N)	183	669	1902	2891	1930	1246	237	117	100	
Mortality/SNI	25.7 (47/183)	18.2 (122/669)	13.1 (250/1902)	13 (376/2891)	15.1 (292/1930)	23.1 (288/1246)	29.1 (69/237)	29.1 (34/117)	35 (35/100)	<0.0001
Mortality	17.5 (32/183)	10.5 (70/669)	7.4 (140/1902)	8.1 (233/2891)	9.5 (183/1930)	15.3 (191/1246)	19 (45/237)	18 (21/117)	25 (25/100)	<0.0001
SNI	16.3 (29/178)	11.1 (73/656)	8.7 (161/1856)	7.4 (207/2816)	8.8 (166/1879)	13.8 (166/1207)	16.4 (38/232)	20.9 (24/115)	22.2 (22/99)	<0.0001
BPD	60.7 (94/155)	52.1 (313/601)	47.5 (838/1764)	48.4 (1299/2683)	49.3 (871/1767)	56.7 (606/1069)	64.1 (127/198)	60.8 (59/97)	67.5 (52/77)	<0.0001
PDA	63.3 (114/180)	64.1 (427/666)	53.5 (1014/1896)	55 (1584/2878)	54.6 (1046/1917)	61.5 (763/1241)	67.5 (158/234)	70.1 (82/117)	62.6 (62/99)	<0.0001

* Data are presented as % (n/N). ** The reported *p*-Values are based on comparisons among the weight change groups using the Chi-square test. BPD = bronchopulmonary dysplasia; N = number of eligible infants; n = number of infants with outcome data; PDA = patent ductus arteriosus; SNI = severe neurological injury. ** The denominator may vary for different characteristics due to missing data.

**Table 4:** Association between outcomes and weight change (% of birth weight) at day 3 from birth.

	Model with Adjustment *		
Outcomes	Delta_W ‡ β (95%CI **) × 10^−3^	Delta_W × delta_W ‡ α (95%CI **) × 10^−3^	Minimum point (C) ‡
Mortality/SNI	15.8 (2.9, 28.7)	0.6 (0.2, 0.99)	−13.16 (−13.18, −13.15)
Mortality	14.8 (3.6, 26)	0.5 (0.1, 0.9)	−14.8 (−14.82, −14.78)
SNI	16.5 (3.8, 29.2)	0.7 (0.3, 1.1)	−11.78 (−11.80, −11.77)
BPD	7.8 (−0.8, 16.4)	0.4 (−0.01, 0.8)	NA †
PDA	1.8 (−6, 9.5)	0.4 (−0.03, 0.8)	NA †

* Multivariable logistic model adjusted for GA, SGA, sex, SNAP-II score, outborn status, Cesarean section, antenatal steroid use, and chest compressions at birth with GEE approach to account for the clustering of infants within NICUs. ‡ Delta W = weight change between birth and day three (% of birth weight); Delta_W × delta_W = quadratic term of the weight change; β × 10^−3^ and α × 10^−3^ = estimated coefficient of Delta_W and Delta_W × Delta_W in the model, respectively. Min point (C) = estimated minimum point (C = −β/(2α)) at which the fitted curve reaches the minimum. ** 95% CI = 95% confidence interval, the estimated coefficient was significantly different from 0 if the 95% CI did not include 0. † NA: α being not significant implied that no ‘U’-shaped relationship between the outcome and the weight change at day three was observed, and therefore the minimum point was not available. BPD = bronchopulmonary dysplasia; PDA = patent ductus arteriosus; SNAP-II = Score for Neonatal Acute Physiology II; SNI = severe neurological injury.

**Table 5:** Comparisons of outcomes (frequency and percentage) depending on weight change (% of birth weight) at day three of age.

Outcomes	Weight Change at Day 3 from Birth (% of Weight at Birth)			*p*-Value *
	<−15	[−15, −9)	≥−9	
Infants (N)	852	2481	5942	
Mortality/SNI, %(n/N)	19.8 (169/852) ^a^	12.7 (316/2481)	17.3 (1028/5942) ^b^	<0.0001
Mortality, %(n/N)	12.0 (102/852) ^a^	7.4 (183/2481)	11.0 (655/5942) ^b^	<0.0001
	**<−13**	**[−13, −9)**	**≥−9**	
SNI, %(n/N)	11.5 (162/1409) ^d^	7.3 (135/1845)	10.2 (589/5784) ^c^	<0.0001

* The reported *p*-values are based on comparisons among the three groups using the Chi-square test. Pairwise comparisons were also conducted: a: *p* < 0.001, comparison between <−15 and −15, −8.9 groups; b: *p* < 0.001, comparison between ≥−9 and −15, −8.9 groups; c: *p* < 0.001, comparison between ≥ −9 and −15, −8.9 groups; d: *p* < 0.001, comparison between <−13 and −13, −8.9 groups. SNI = severe neurological injury.

**Table 6:** Comparisons of outcomes depending on weight change (% of birth weight) at day three of age.

Outcomes	Weight Change at day 3 (% of Weight at Birth)		
	<−15	[−15 to −9)	≥−9
Mortality/SNI *	1.5 (1.3, 1.8)	1 (ref)	1.4 (1.3, 1.6)
Mortality *	1.7 (1.4, 2.1)	1 (ref)	1.6 (1.3, 1.9)
Mortality/SNI **	1.4 (1.1, 1.7)	1 (ref)	1.2 (1.03, 1.5)
Mortality **	1.4 (1.1, 1.7)	1 (ref)	1.3 (1.02, 1.7)
	**<−13**	**[−13 to −9)**	**≥−9**
SNI *	1.6 (1.3, 1.9)	1 (ref)	1.5 (1.2, 1.7)
SNI **	1.4 (1.2, 1.8)	1 (ref)	1.3 (1.06, 1.5)

* OR (95% CI) = raw odds ratio of outcomes for the region of weight change at day 3 (vs. ref) based on logistic regression model without adjustment. ** AOR (95% CI) adjusted odds ratio of outcomes for the region of weight change at day 3 (vs. ref) based on multivariable logistic regression model with generalized estimating equation (GEE) approach to account for the clustering of infants within NICUs, adjusted for gestational age, small for gestational age, sex, SNAP-II score, outborn status, Cesarean section, antenatal steroid use, and chest compressions at birth. CI = confidence interval; SNI = Severe neurological injury; ref = reference group.

## Data Availability

The data presented in this study are available on request from the corresponding author after approval from the local Research Ethics Board.

## Abbreviations

BPD	bronchopulmonary dysplasia
CI	95% confidence interval
CNN	Canadian Neonatal Network
delta_W3	weight change from birth to day three of postnatal age
GA	gestational age
GEE	generalized estimating equation
IQR	interquartile range
IVH	intraventricular hemorrhage
NICU	neonatal intensive care unit
PDA	patent ductus arteriosus
SNAP-II	score for neonatal acute physiology II
SGA	small for gestational age
SNI	severe neurological injury

## References

[1] Modi N. (2004). Management of fluid balance in the very immature neonate. Arch. Dis. Child Fetal Neonatal Ed..

[2] Chow J.M., Douglas D. (2008). Fluid and Electrolyte Management in the Premature Infant. Neonatal Netw..

[3] Rochow N., Raja P., Liu K., Fenton T., Landau-Crangle E., Göttler S., Jahn A., Lee S., Seigel S., Campbell D. (2016). Physiological adjustment to postnatal growth trajectories in healthy preterm infants. Pediatr. Res..

[4] Dalton J., Dechert R., Sarkar S. (2014). Assessment of Association between Rapid Fluctuations in Serum Sodium and Intraventricular Hemorrhage in Hypernatremic Preterm Infants. Am. J. Perinatol..

[5] Oh W., Poindexter B.B., Perritt R., Lemons J.A., Bauer C.R., Ehrenkranz R.A., Stoll B.J., Poole K., Wright L.L. (2005). Association between Fluid Intake and Weight Loss during the First Ten Days of Life and Risk of Bronchopulmonary Dysplasia in Extremely Low Birth Weight Infants. J. Pediatr..

[6] Stephens B.E., Gargus R.A., Walden R.V., Mance M., Nye J., McKinley L., Tucker R., Vohr B.R. (2008). Fluid regimens in the first week of life may increase risk of patent ductus arteriosus in extremely low birth weight infants. J. Perinatol..

[7] Bell E.F., Acarregui M.J. (2014). Restricted versus liberal water intake for preventing morbidity and mortality in preterm infants. Cochrane Database Syst. Rev..

[8] Hartnoll G., Bétrémieux P., Modi N. (2000). Randomised controlled trial of postnatal sodium supplementation on body composition in 25 to 30 week gestational age infants. Arch. Dis. Child.-Fetal Neonatal Ed..

[9] Segar J. (2020). A physiological approach to fluid and electrolyte management of the preterm infant: Review. J. Neonatal-Perinat. Med..

[10] The Canadian Neonatal NetworkTM. http://www.canadianneonatalnetwork.org/portal/.

[11] Canadian Neonatal Network Canadian Neonatal Network Abstractor’s Manual. v 2.1.2. 2014; pp. 1–94. www.canadianneonatalnetwork.org.

[12] Seidlitz W., Chan P., Yeh S., Musrap N., Lee S.K. (2017). Internal Audit of the Canadian Neonatal Network Data Collection System. Am. J. Perinatol..

[13] Sauve R. (2001). Routine screening cranial ultrasound examinations for the prediction of long term neurodevelopmental outcomes in preterm infants. Paediatr. Child Health.

[14] Jobe A.H., Bancalari E. (2001). Bronchopulmonary dysplasia. Am. J. Respir. Crit. Care Med..

[15] Kramer M.S., Platt R.W., Wen S.W., Joseph K.S., Allen A., Abrahamowicz M., Blondel B., Breart G., for the Fetal/Infant Health Study Group of the Canadian Perinatal Surveillance System (2001). A New and Improved Population-Based Canadian Reference for Birth Weight for Gestational Age. Pediatrics.

[16] Richardson D.K., Corcoran J.D., Escobar G.J., Lee S.K. (2001). SNAP-II and SNAPPE-II: Simplified newborn illness severity and mortality risk scores. J. Pediatr..

[17] Verma R.P., Shibli S., Fang H., Komaroff E. (2009). Clinical determinants and utility of early postnatal maximum weight loss in fluid management of extremely low birth weight infants. Early Hum. Dev..

[18] Barnette A.R., Myers B.J., Berg C.S., Inder T.E. (2010). Sodium intake and intraventricular hemorrhage in the preterm infant. Ann. Neurol..

[19] Lim W.-H., Lien R., Chiang M.-C., Fu R.-H., Lin J.-J., Chu S.-M., Hsu J.-F., Yang P.-H. (2010). Hypernatremia and grade III/IV intraventricular hemorrhage among extremely low birth weight infants. J. Perinatol..

[20] Lee H.J., Lee B.S., Do H.-J., Oh S.-H., Choi Y.-S., Chung S.-H., Kim E.A.-R., Kim K.-S. (2015). Early Sodium and Fluid Intake and Severe Intraventricular Hemorrhage in Extremely Low Birth Weight Infants. J. Korean Med. Sci..

[21] Verma R., Shibly S., Fang H., Pollack S. (2015). Do early postnatal body weight changes contribute to neonatal morbidities in the extremely low birth weight infants. J. Neonatal-Perinat. Med..

[22] Aksoy H.T., Güzoğlu N., Eras Z., Gökçe I.K., Canpolat F.E., Uraş N., Oğuz S.S. (2019). The association of early postnatal weight loss with outcome in extremely low birth weight infants. Pediatr. Neonatol..

[23] Wadhawan R., Oh W., Perritt R., Laptook A.R., Poole K., Wright L.L., Fanaroff A.A., Duara S., Stoll B.J., Goldberg R. (2007). Association between early postnatal weight loss and death or BPD in small and appropriate for gestational age extremely low-birth-weight infants. J. Perinatol..

[24] NICHD Neonatal Research Network. https://neonatal.rti.org/.

[25] Poindexter B.B., Ehrenkranz R.A., Stoll B.J., Wright L.L., Poole W.K., Oh W., Bauer C.R., Papile L.-A., Tyson J.E., Carlo W.A. (2004). Parenteral Glutamine Supplementation Does Not Reduce the Risk of Mortality or Late-Onset Sepsis in Extremely Low Birth Weight Infants. Pediatrics.

[26] Lorenz J.M., Kleinman L.I., Ahmed G., Markarian K. (1995). Phases of fluid and electrolyte homeostasis in the extremely low birth weight infant. Pediatrics.

[27] Lorenz J., Polin R., Yoder M. (2014). Fluid & electrolyte management in newborn intensive care unit. Workbook in Practical Neonatology.

[28] Iacobelli S., Guignard J.P., Polin R., Abman S., Rowitch D., Benitz W. (2016). Physiology of the urinary diluting mechanism. Fetal and Neonatal Physiology.

[29] Barnett H.L., Vesterdal J., McNamara H., Lauson H.D. (1952). Renal water excretion in premature infants 12. J. Clin. Investig..

[30] Vieux R., Hascoet J.-M., Merdariu D., Fresson J., Guillemin F. (2010). Glomerular Filtration Rate Reference Values in Very Preterm Infants. Pediatrics.

[31] O’Brien F., Walker I.A. (2013). Fluid homeostasis in the neonate. Pediatr. Anesth..

[32] Hammarlund K., Sedin G. (1979). Transepidermal loss in newborn infants III Relation to gestational age. Acta Paediatr..

[33] Hammarlund K., Sedin G., Strömberg B. (1982). Transepidermal water loss in newborn infants. VII. Relation to post-natal age in very pre-term and full-term appropriate for gestational age infants. Acta Paediatr. Scand..

[34] Ågren G., Sjörs G.S.J. (1998). Transepidermal water loss in infants born at 24 and 25 weeks of gestation. Acta Paediatr..

[35] Gawlowski Z., Aladangady N., Coen P.G. (2006). Hypernatraemia in preterm infants born at less than 27 weeks gestation. J. Paediatr. Child Health.

